# Exposure time independent summary statistics for assessment of drug dependent cell line growth inhibition

**DOI:** 10.1186/1471-2105-15-168

**Published:** 2014-06-05

**Authors:** Steffen Falgreen, Maria Bach Laursen, Julie Støve Bødker, Malene Krag Kjeldsen, Alexander Schmitz, Mette Nyegaard, Hans Erik Johnsen, Karen Dybkær, Martin Bøgsted

**Affiliations:** 1 Department of Haematology, Aalborg University Hospital, Aalborg, Denmark; 2 Department of Biomedicine, Aarhus University, Aarhus, Denmark; 3 Department of Mathematical Sciences, Aalborg University, Aalborg, Denmark

**Keywords:** Dose response experiments, NCI60, Doxorubicin, Mathematical modelling, Differential equation modelling, Nonlinear regression, Isotonic regression, Bootstrap, Parametric bootstrap

## Abstract

**Background:**

*In vitro* generated dose-response curves of human cancer cell lines are widely used to develop new therapeutics. The curves are summarised by simplified statistics that ignore the conventionally used dose-response curves’ dependency on drug exposure time and growth kinetics. This may lead to suboptimal exploitation of data and biased conclusions on the potential of the drug in question. Therefore we set out to improve the dose-response assessments by eliminating the impact of time dependency.

**Results:**

First, a mathematical model for drug induced cell growth inhibition was formulated and used to derive novel dose-response curves and improved summary statistics that are independent of time under the proposed model. Next, a statistical analysis workflow for estimating the improved statistics was suggested consisting of 1) nonlinear regression models for estimation of cell counts and doubling times, 2) isotonic regression for modelling the suggested dose-response curves, and 3) resampling based method for assessing variation of the novel summary statistics. We document that conventionally used summary statistics for dose-response experiments depend on time so that fast growing cell lines compared to slowly growing ones are considered overly sensitive. The adequacy of the mathematical model is tested for doxorubicin and found to fit real data to an acceptable degree. Dose-response data from the NCI60 drug screen were used to illustrate the time dependency and demonstrate an adjustment correcting for it. The applicability of the workflow was illustrated by simulation and application on a doxorubicin growth inhibition screen. The simulations show that under the proposed mathematical model the suggested statistical workflow results in unbiased estimates of the time independent summary statistics. Variance estimates of the novel summary statistics are used to conclude that the doxorubicin screen covers a significant diverse range of responses ensuring it is useful for biological interpretations.

**Conclusion:**

Time independent summary statistics may aid the understanding of drugs’ action mechanism on tumour cells and potentially renew previous drug sensitivity evaluation studies.

## Background

An essential part of discovery and development of anticancer drugs is to assess the induced growth inhibition in a biologically broad range of tumour derived cell lines by dose-response experiments [1,2]. The three large cell line screens NCI60 [3,4], JFCR39 [5,6], and CMT1000 [2,7] are among the most well-known high throughput cell line drug screens.

The approach used in CMT1000 and several other studies [8-10] is currently the standard approach for conducting dose-response experiments. The experiments are performed by challenging exponentially growing cell lines with a serial dilution of drug concentrations and estimating growth inhibition by relative cell counts between the treated and untreated cell line. Then, a summary statistic of drug efficiency ${\text{GI}}_{50}^{R}$ (50% growth inhibition) is obtained by estimating the concentration at which the relative cell count is 50% after a fixed period of time. Hence, neither drug exposure time nor varying cell line growth rates are considered.

**Figure 1:**
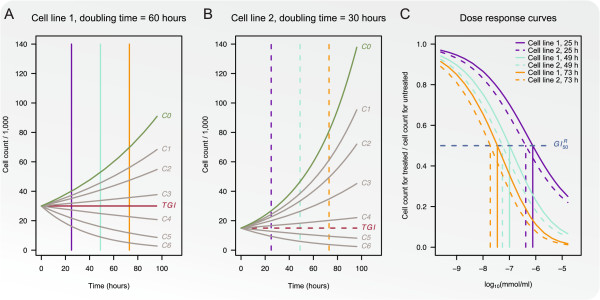
**Illustration of growth inhibition assessed by relative cell counts.** Panels **A** and **B** show growth curves for two cell line models with doubling times of 60 and 30 hours, respectively. The cell line models are treated with 6 increasing concentrations *C*1,…,*C*6 and growth curves for each concentration are shown. The red line illustrates total growth inhibition (*TGI*). Dose-response curves calculated by relative cell counts for time points 25, 49, and 73 hours are shown in Panel **C**.

The method is easily comprehended and implemented, however, as illustrated in Figure 1 this assessment of growth inhibition leads to summary statistics that are difficult to interpret. Panels A and B illustrate generated growth curves for two cell line models with doubling times 60 and 30 hours, respectively. The cell line models are treated with 6 increasing concentrations *C*1,…,*C*6 of a potent drug for which the effect is assumed constant through time, resulting in 6 growth/decay curves. For concentration *C*4 cell line model 1 is in the decay phase and cell line model 2 is in the growth phase suggesting that cell line model 1 is the more sensitive of the two.

Panel C illustrates dose-response curves calculated at three time points: 25, 49, and 73 hours, for the two cell line models. Because of the fast growth rate of cell line model 2, the summary statistic ${\text{GI}}_{50}^{R}$ is obtained at a lower concentration for this cell line model than for cell line model 1 for each of the three time points. This indicates that cell line model 2 is evaluated as the more sensitive of the two. Hence, this assessment of growth inhibition generates summary statistics that are incomparable between cell lines with different growth rates.

The dose-response experiments performed for the NCI60 and JFCR39 screens are summarised by comparing net differences between cell counts at observation time and the initial cell counts for the treated and untreated cell lines. As we illustrate later this method only partially solves the problem of growth rate dependency.

The concept behind the present work is that modelling the growth of a cell line exposed to a drug by a simplified differential equation will allow us to derive dose-response curves and summary statistics that are independent of time under the proposed model. For estimation of the improved summary statistics a statistical workflow is suggested consisting of 1) pre-processing of absorbance measurements to account for multiplicative errors originating from e.g. cell line seeding [11] and correcting for background absorbance caused by the drug [12], 2) isotonic regression for modelling the dose-response curve which is robust against outliers and model misspecifications [13,14], and 3) a bootstrap method for estimation of confidence intervals for summary statistics [9]. We also aim to illustrate a transformation of the model used in the cell line screen NCI60, which accounts for each cell line’s doubling time and enables a reanalysis of existing dose-response data.

Finally, the adequacy of the differential equation for modelling real data is tested using a doxorubicin screen. The screen is also used to investigate the applicability of the proposed statistical analysis workflow by providing variance estimates for obtained exposure time independent summary statistics.

## Methods

### The mathematical model

**Figure 2:**
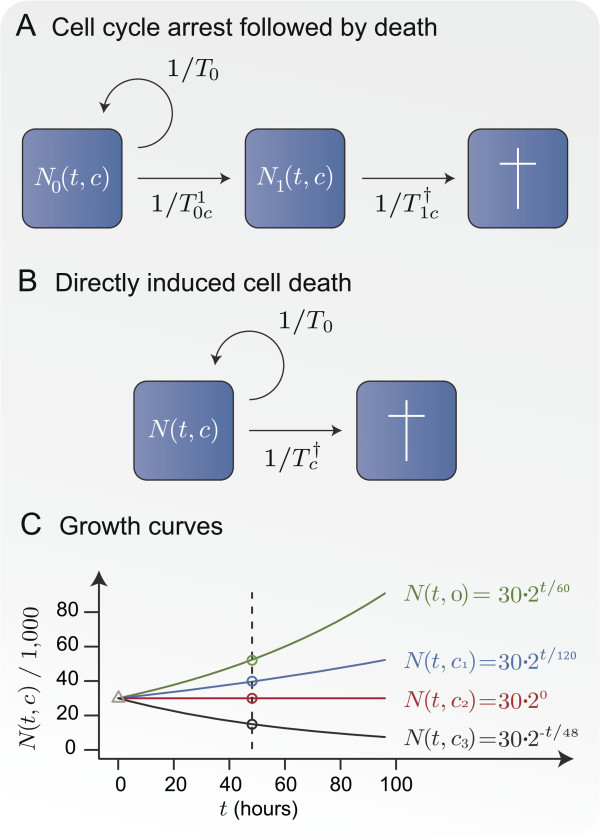
**Illustration of the proposed compartment models.** Panel **A** illustrates a compartment model where the drug is assumed to induce cell cycle arrest with halving time $T_{0c}^{1}$ and for cells in cell cycle arrest the death rate is assumed exponential with halving time $T_{1c}^{†}$. Panel **B** illustrates a simplified compartment model in which the drug is assumed to induce cell death with halving time $T_{c}^{†}$. In both models the cell line grows with doubling time *T*_0_. Panel **C** illustrates growth curves according to Model B for a cell line with *T*_0 _= 60 hours and *N*_0 _= 30,000 cells treated with three different concentrations of a potent drug. The concentrations correspond to the summary statistics *GI*_50 _= *c*_1_, *TGI *=*c*_2_, and *LC*_48 _= *c*_3_.

To analyse dose-response experiments rigorously we formulate a model of how the growth of a cell line is influenced by a given drug. The growth inhibition is modelled by the compartment models illustrated in Figures 2A and B. Panel A shows a compartment model for drugs that induce cell cycle arrest followed by death. For a cell line treated with drug concentration *c *≥ 0 the number of unaffected cells at time *t* is denoted *N*_0 _(*t*,*c*). The growth of this cell population is assumed exponential with doubling time *T*_0 _or equivalently a growth rate of 1/*T*_0_. The concentration dependent rate for cells going into cell cycle arrest is likewise assumed exponential with halving time $T_{0c}^{1}>0$, and *N*_1 _(*t*,*c*) denotes the cell count for this population. Finally, the death rate is assumed exponential and concentration dependent with halving time $T_{1c}^{†}>0$.

We focus on dose-response experiments where the cell count is estimated indirectly by cell metabolism. However, cells in cell cycle arrest have a very low metabolism and such cells are indistinguishable from dead cells in these experiments [15]. Because of this we use the simplified compartment model illustrated in Figure 2B for drugs that induce cell cycle arrest as well as drugs that lead directly to cell death. In this model *N *(*t*,*c*) denotes the cell count of metabolising cells at time *t* for a cell line treated with drug concentration *c* ≥ 0. The growth rate is assumed to be exponential with doubling time *T*_0_. Similarly, the death rates are assumed to be exponential with halving times $T_{c}^{†}>0$ that decrease concurrently with increasing drug concentrations *c*. For drugs that induce cell cycle arrest the halving time $T_{c}^{†}>0$ is equal to the rate with which cells go into cell cycle arrest when treated with drug concentration *c*. Compartment model B gives rise to the following differential equation model

(1) $$\begin{array}{l} \frac{\text{dN}(t,c)}{\text{dt}}=\frac{\text{log}2}{T_{0}}N(t,c)-\frac{\text{log}2}{T_{c}^{†}}N(t,c). \end{array}$$

The differential equation has the following solution

(2) $$\begin{array}{l} N(t,c)=N_{0}2^{t/T_{0}-t/T_{c}^{†}}=N_{0}2^{t/T_{c}}, \end{array}$$

where the initial condition *N*_0 _= *N *(0,*c*), with *c * dropped for short, denotes the cell count at *t *= 0,

(3) $$\begin{array}{l} \frac{1}{T_{c}}=\frac{1}{T_{0}}-\frac{1}{T_{c}^{†}}, \end{array}$$

and *T*_*c *_corresponds to the net observed doubling or halving time at concentration *c*.

The posed differential equation model can be summarised by the following statistics

(4) $$\begin{array}{l} {\text{GI}}_{50}:\;\frac{T_{0}}{T_{c}}=\frac{1}{2}\;\text{TGI}:\;T_{0}=T_{c}^{†}\;{\text{LC}}_{t}:\;\frac{1}{T_{c}}=-\frac{1}{t}, \end{array}$$

where *GI*_50 _(50% growth inhibition) denotes the concentration at which the cell line grows with a doubling time twice as long as the same cell line untreated, *TGI* (total growth inhibition) denotes the concentration at which the cell line has no net growth, and *LC*_*t *_(lethal concentration *t*) denotes the concentration at which the cell count decays with a halving time of *t* hours. For example *LC*_48_ is the concentration at which *N *(48,*c*) = *N*_0_/2.

The growth inhibition induced by these drug concentrations is illustrated in Figure 2C for a cell line model with doubling time *T*_0 _= 60 hours and *N*_0 _= 30,000. At the concentration corresponding to *GI*_50_ the doubling time for the cell line is doubled to 120, *TGI* the halving time $T_{c}^{†}=T_{0}=60$ such that the growth of the cell line is completely halted, and *LC*_48 _the halving time for the cell line is 48 hours.

This leads us to suggest the following *growth* based dose-response model denoted by *G* for evaluating a dose-response experiment

(5) $$\begin{array}{l} G(t,c)=\left\{\begin{array}{ll} \frac{T_{0}}{T_{c}} & \text{if}N(t,c)\geq N_{0}\\ \frac{1}{T_{c}} & \text{otherwise.} \end{array}\right) \end{array}$$

It is noteworthy that the *G*-model is independent of the duration of the dose-response experiment. The model is summarised by the statistics *GI*_50_, *TGI*, and *LC*_48 _at which the *G *(*t*,*c*) equals 0.5, 0, and -1/48, respectively. In general we define *GI*_*x *_and *LC*_*t *_to be the concentrations where *G *(*t*,*c*) = (100-*x*)/100 and *G *(*t*,*c*) = -1/*t*.

The cell line screens NCI60 [3,4] and JFCR39 [5,6] apply an alternative dose-response model denoted by *D*, which is based on net *differences* between the cell count at time *t*, *N *(*t*,*c*), and the initial cell count, *N*_0_. The model is defined as

(6) $$\begin{array}{llll} D(t,c) & = & \left\{\begin{array}{ll} \frac{N(t,c)-N_{0}}{N(t,0)-N_{0}} & \text{if}N(t,c)\geq N_{0}\\ \frac{N(t,c)-N_{0}}{N_{0}} & \text{otherwise} \end{array}\right) & \\ & = & \left\{\begin{array}{ll} \frac{2^{t/T_{c}}-1}{2^{t/T_{0}}-1} & \text{if}N(t,c)\geq N_{0}\\ 2^{t/T_{c}}-1 & \text{otherwise}. \end{array}\right) & \end{array}$$

The cell counts which the *D*-model is based upon are illustrated by the triangle and circles in Figure 2C for *t *= 48 hours. For this model *x% *growth inhibition $GI_{x}^{D}$ and *y% *lethal concentration $LC_{y}^{D}$ are attained at concentrations *c*_1_ and *c*_2_ where *D*(*t*,*c*_1_) = (100-*x*)/100 and *D *(*t*,*c*_2_) = -(100-*y*)/100. The dose-response model is usually summarised for a fixed *t *by ${\text{GI}}_{50}^{D}$, ${\text{LC}}_{50}^{D}$, and *TGI*^*D *^the latter of which is attained at the concentration *c *where *D *(*t*,*c*) = 0.

The large cell line screen CMT1000 [2,7] utilises another commonly used dose-response model based on *relative* cell counts which is defined as

(7) $$\begin{array}{llll} R(t,c) & = & \frac{N(t,c)/N_{0}}{N(t,0)/N_{0}}=\frac{N(t,c)}{N(t,0)} & \\ & = & 2^{\left(\frac{1}{T_{c}}-\frac{1}{T_{0}}\right)\cdot t}=2^{-t/T_{c}^{†}}. & \end{array}$$

The cell counts which the *R*-model is based upon are illustrated by circles in Figure 2C for *t *= 48 hours. For this model *x%* growth inhibition $GI_{x}^{R}$ is attained at concentration *c* where *R *(*t*,*c*) = (100-*x*)/100. The *R*-model is usually summarised by ${\text{GI}}_{25}^{R}$, ${\text{GI}}_{50}^{R}$, and ${\text{GI}}_{75}^{R}$.

For a fixed *t*, the graph of a dose-response model, say *G*, {(*c*,*G *(*t*,*c*)):*c* > 0} is denoted the dose-response curve of *G*. As the *D*- and *R*-models suggest, the corresponding dose-response curves are dependent on the time *t*, whereas the dose-response curve of *G* is not.

Notice it is possible to define a fourth summary statistic *AUC*_*q *_(area under curve) which is the area above a specified value *q* and below the dose-response curve [16]. Thus, for the dose-response models *G* and *D*, *AUC*_0 _is the area under the dose-response curve for which the cell count is still increasing with time.

It is possible to circumvent the time dependency of models *D* and *R* by letting the drug exposure time *t* equal the cell line specific doubling time *T*_0_ or a multiple hereof, i.e. *t *= *kT*_0_. Furthermore, the summary statistics for the *G*-model are then related with the *D*-model by

$$\begin{array}{llll} {\text{GI}}_{50} & = & GI_{x}^{D},\;\text{where}\;x=\frac{2^{\frac{1}{2}k}-1}{2^{k}-1}\cdot 100 & \\ \text{TGI} & = & \text{TG}I^{D} & \\ {\text{LC}}_{48} & = & LC_{x}^{D},\;\text{where}\;x=\left(2^{-\frac{1}{48}kT_{0}}-1\right)\cdot 100 & \end{array}$$

and for the *R*-model by

$$\begin{array}{llll} {\text{GI}}_{50} & = & GI_{x}^{R},\;\text{where}\;x=2^{-\frac{1}{2}k}\cdot 100 & \\ \text{TGI} & = & GI_{x}^{R},\;\text{where}\;x=2^{-k}\cdot 100 & \\ {\text{LC}}_{48} & = & GI_{x}^{R},\;\text{where}\;x=2^{-\frac{1}{48}{\text{kT}}_{0}-k}\cdot 100. & \end{array}$$

Even if the drug exposure time is not *kT*_0_ it is still possible to obtain an estimate of the *G*-model by the following transformation of the *D*-model

(8) $$\begin{array}{l} G(t,c)=\left\{\begin{array}{ll} \frac{T_{0}}{t}\underset{2}{\text{log}}\left(\left(2^{\frac{t}{T_{0}}}-1\right)D(t,c)+1\right) & \text{if}D(t,c)\geq 0\\ \frac{1}{t}\underset{2}{\text{log}}\left(D(t,c)+1\right) & \text{otherwise.} \end{array}\right) \end{array}$$

Similarly, an estimate of the *G*-model can be obtained by the following transformation of the *R*-model

(9) $$\begin{array}{l} G(t,c)=\left\{\begin{array}{ll} 1+\frac{T_{0}}{t}\underset{2}{\text{log}}\left(R(t,c)\right) & \text{if}\frac{t}{T_{0}}>\left|\underset{2}{\text{log}}\left(R(t,c)\right)\right|\\ \frac{1}{T_{0}}+\frac{1}{t}\underset{2}{\text{log}}\left(R(t,c)\right) & \text{otherwise.} \end{array}\right) \end{array}$$

Note, however, that both transformations require access to the cell line specific doubling time *T*_0_.

### Estimation of cell count

Absorbance measurements can be utilised as surrogates for the cell count *N*(*t*,*c*) and thereby used to estimate the three dose-response models. This is generally done using an MTS assay (3-(4,5-dimethylthiazol-2-yl)-5-(3-carboxymethoxyphenyl)-2-(4-sulfophenyl)-2H-tetrazolium) that exploits the mitochondrial reduction of tetrazolium to an aqueous soluble formazan product by the dehydrogenase enzyme in viable cells at 37°C. The amount of produced formazan is directly proportional to the cell count *N *(*t*,*c*) and can be quantified colourimetrically by measuring absorbance at 492 nm [12].

**Figure 3:**
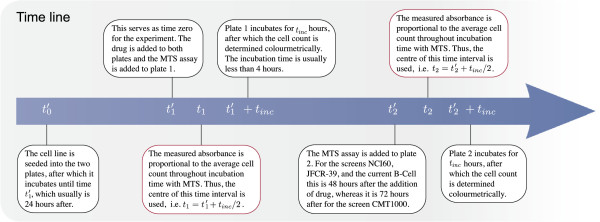
**Time line for dose-response experiments.** The red boxes mark the two time points that are used in the statistical analysis.

The time line for dose-response experiments utilising such assays is outlined in Figure 3. At time $t_{0}^{\prime }$ each cell line is seeded into two 96 well plates in which they incubate until time $t_{1}^{\prime }=0$ where *C* decreasing concentrations of the tested drug are added to each plate in *L* replicates. This time point marks the start of the dose-response experiment. For plate 1 the MTS assay is added immediately after drug exposure i.e. at time $t_{1}^{\prime }$ and for plate 2 the assay is added at time $t_{2}^{\prime }$ a pre-specified time after the addition of drug. Following the addition of the reagent each plate incubates for a fixed time *t*_*inc *_after which the absorbance is measured. The metabolic reduction occurs from the instant the reagent is added until the absorbance is measured, thus the amount of formazan is related to the cell count during this time interval. Since the number of living cells may differ substantially throughout this period, the measured absorbance is assumed to estimate the cell count at the centre of the interval, i.e. at time $t_{1}=t_{1}^{\prime }+t_{\text{inc}}/2$ and $t_{2}=t_{2}^{\prime }+t_{\text{inc}}/2$.

By seeding cells in an appropriate medium at predetermined concentrations the relationship between the cell count and the absorbance measure can be assumed linear [12], i.e.

(10) $$\begin{array}{l} \alpha_{\text{ti}}=\gamma N(t,c_{i}), \end{array}$$

where *γ *is a proportionality factor and *α*_*ti *_is the absorbance at time *t*, for a cell line exposed to drug concentration *c*_*i*_, *i *= 1,…,*I *where 0 = *c*_0 _<*c*_1 _< ⋯ <*c*_*I*_. The proportionality factor is cell line specific due to individual capabilities of reducing tetrazolium into the coloured formazan product.

In order to ensure reproducible results the experiment is repeated *K* times for each cell line. The measured absorbance level for the *l*’th well, treated with concentration *c*_*i*_, within the *k*’th cell line replicate, is assumed equal to

(11) $$\begin{array}{ll} \;Y_{\text{ktil}} & =\delta_{\text{kt}}\alpha_{\text{ti}}+\beta_{\text{kt}}+\epsilon_{\text{ktil}},\;\\ k=1,\ldots ,K;\;t & =t_{1},t_{2};\;i=0,1,\ldots ,I;\;l=1,\ldots ,L,\; \end{array}$$

where *δ*_*kt *_is the inter-plate variation assumed to be multiplicative, *β*_*kt *_is the plate specific background absorbance which is assumed to be additive, and, finally, the technical variation *ε*_*ktil *_is assumed to be additive and normally distributed with mean zero and following a heteroscedastic variance model $|\delta_{\text{kt}}\alpha_{\text{ti}}+\beta_{\text{kt}}{|}^{2\xi }\sigma_{\beta }^{2}$.

### Statistical analysis workflow

The proposed statistical workflow has been implemented in the statistical software R version 3.0.1. The parameter estimation is performed using the function gnls from the package nlme[17]. Isotonic regression is implemented by the function isoreg from the library stats, and the area under the curve is calculated by the function trapz from the library pracma.

#### ***Model-based pre-processing***

Conventionally pre-processing is performed through the following steps: 1) the background wells containing only medium are used to measure the plate specific background absorbance, which are averaged and subtracted from the absorbance measures of all other wells, 2) normalisation is done indirectly through calculation of growth inhibition by either of the dose-response models *D* or *R*, and 3) summation is done by an average of the obtained values. In this article, we propose a model-based approach for the pre-processing. The pre-processing of the dose-response experiments consists of outlier detection, background correction, normalisation, and summation. These steps are done simultaneously by estimating the coefficients *β*_*kt*_, *δ*_*kt*_, and *α*_*ti *_in absorbance model (11). In order to do this, the absorbance model is reformulated as a nonlinear regression model:

$$Y_{\text{ktbil}}=\phi_{1\text{tki}}\phi_{2\text{tki}}s_{b}+\phi_{3\text{tki}}+\beta_{\text{ktil}},$$

where *s*_*b*_, *b *= 1,…,*n*_*kt *_, is an indicator variable equal to 0 if *Y*_*ktbil *_is a background measurement and 1 otherwise, *ϕ*_*tki *_= *A*_*tki*_*β*_*t*_, and

The nonlinear regression model corresponds to the formalism in Chapter 7 of [17] and can be estimated by the methods herein. The components of the heteroscedastic variance is estimated by an iteratively reweighted scheme, see page 207 of [17]. The *I *+ 1 coefficients ${\hat{\alpha }}_{\text{ti}}$ are the summarised absorbance measures for each concentration *c*_*i*_. Since negative absorbance measures are meaningless, all absorbance estimates below a pre-specified value are replaced by this value. We use the value 0.025 as the cut point in the current study.

One of the favourable features of the model-based approach to pre-processing dose-response experiments is outlier detection based on residuals. The residuals are the difference between the observed values and the values estimated by the regression model. First, the regression model is fitted to all data. Absorbance measures with residuals greater than a pre-specified number of standard deviations are regarded as outliers and removed. Based on the remaining absorbance measures the model is fitted again and outliers are detected and removed. This process is iterated until no outliers are detected or until a pre-specified maximum number of iterations is reached. In the current study we use 3 standard deviations and iterate the process twice.

#### ***Estimation of cell line doubling times***

When estimating the dose-response curve by the *G*-model (5) we need estimates of *T*_0 _and the *T*_*c*_’s. This can be done by estimating the coefficients *N*_0_, *T*_0_, and $T_{c}^{†}$ in the solution to the proposed differential equation (2). The resulting estimates of the absorbance measures ${\hat{\alpha }}_{\text{tc}}$ obtained by the pre-processing method are used as estimates of *γN *(*t*,*c*). Thus we consider the following nonlinear model

(12) $$\begin{array}{l} {\hat{\alpha }}_{\text{ti}}=\alpha_{0}2^{\frac{t}{T_{0}}-s_{i}\frac{t}{T_{c_{i}}^{†}}}, \end{array}$$

where *α*_0_ is an estimate of *γN*_0_, *s*_*i *_is an indicator function equal to 0 when *i *= 0 and 1 otherwise. This nonlinear model is also formulated in concordance with Chapter 7 in [17]. This gives the nonlinear regression function

(13) $$\begin{array}{l} {\hat{\alpha }}_{\text{ti}}=\phi_{1}2^{t\left(\phi_{2}-s_{i}\cdot \phi_{3i}^{2}\right)}+\beta_{\text{ti}}, \end{array}$$

where *ϕ*_*i *_= *A*_*i*_*β*, and

$$\begin{array}{l} \underset{\phi_{i}}{\underset{︸}{\left[\begin{array}{l} \phi_{1}\\ \phi_{2}\\ \phi_{3i} \end{array}\right]}}=\underset{A_{i}}{\underset{︸}{\left[\begin{array}{ll} 1 & 0\\ 0 & 1\\ 0 & 0 \end{array}\right)\overset{{\left(i+2\right)}^{\prime }\text{th}}{\underset{I}{\underset{︸}{\left(\begin{array}{lllll} 0 & \cdots & \cdots & \cdots & 0\\ 0 & \cdots & \cdots & \cdots & 0\\ 0 & \cdots & 010 & \cdots & 0 \end{array}\right]}}}}}\underset{\beta }{\underset{︸}{\left[\begin{array}{l} \alpha_{0}\\ T_{s0}\\ T_{\text{sc}1}^{†}\\ \vdots \\ T_{\text{scI}}^{†} \end{array}\right]}}. \end{array}$$

The additive error term *ε*_*tc *_is assumed to be normally distributed with mean zero and variance $\sigma_{\beta }^{2}$. The estimates of *T*_0_ and $T_{c_{i}}^{†}$ are calculated as ${\hat{T}}_{0}=1/{\hat{T}}_{s0}{\hat{T}}_{c_{i}}^{†}=1/{\hat{T}}_{{\text{sc}}_{i}}^{†\;2}$. The parameterisations *T*_0 _= 1/*T*_*s*0_, $T_{c_{i}}^{†}=1/{\left(T_{{\text{sc}}_{i}}^{†}\right)}^{2}$ are used to ensure that $T_{c_{i}}^{†}$ is positive. Finally, the estimates of the $T_{c_{i}}$’s are obtained as

(14) $${\hat{T}}_{c_{i}}={\left(\frac{1}{{\hat{T}}_{0}}-\frac{1}{{\hat{T}}_{c_{i}}^{†}}\right)}^{-1},$$

for *i *= 1,…,*I*.

#### ***Estimation of the dose-response curve***

In order to make the estimation method robust against outliers and model misspecifications we suggest to use isotonic regression [13,14] for which the dose-response curve is found by the piecewise linear and decreasing function that in square norm is closest to the data. Because the *G*-model is a piecewise smooth function with a singularity at the *TGI*-value a supplementary function is introduced to circumvent biased estimates. First we define the function *Γ *(*t*,*c*) = 1/*T*_*c *_which is an analogue to the *G*-model in (5) without the multiplication by *T*_0_. Then we estimate the function *Γ* at concentrations *c*_*i*_, *i *= 1,…,*I*, by the values ${\hat{\gamma }}_{1},\ldots ,{\hat{\gamma }}_{I}$ which minimises $\overset{I}{\underset{i=1}{\sum }}{\left(\gamma_{i}-1/T_{c}\right)}^{2}$, subject to *γ*_1 _≥ *γ*_2 _≥ ⋯ ≥ *γ*_*I*_. The *Γ*-function is estimated by linear interpolation in the following way

$$\hat{\Gamma }(t,c)=\overset{I-1}{\underset{i=1}{\sum }}\left({\hat{\gamma }}_{i}+\frac{{\hat{\gamma }}_{i+1}-{\hat{\gamma }}_{i}}{c_{i+1}-c_{i}}\left(c-c_{i}\right)\right)I\left(c_{i}\leq c<c_{i+1}\right)$$

and the *G*-model is then estimated by plugging $\hat{\Gamma }$ and ${\hat{T}}_{0}$ into (5)

(15) $$\begin{array}{l} \hat{G}(t,c)=\left\{\begin{array}{ll} {\hat{T}}_{0}\cdot \hat{\Gamma }(t,c) & \text{if}\hat{\Gamma }(t,c)\geq 0\\ \hat{\Gamma }(t,c) & \text{otherwise.} \end{array}\right) \end{array}$$

The dose-response model *D* is similarly estimated pointwise by (6) with cell counts $\hat{N}(t,c)$ and ${\hat{N}}_{0}$ estimated by the absorbance measures ${\hat{\alpha }}_{\text{tc}}$ and ${\hat{\alpha }}_{0}$. The approach used for the *G*-model is also recommended for estimating the dose-response curve for the *D*-model which also is a piecewise smooth function with a singularity at the *TGI*-value. In this case the function *Γ *(*t*,*c*) is replaced by the function *Δ*(*t*,*c*) = (*N*(*t*,*c*)-*N*_0_)/*N*_0_, and the *D*-model is subsequently estimated by

(16) $$\begin{array}{l} \hat{D}(t,c)=\left\{\begin{array}{ll} \frac{{\hat{N}}_{0}}{\hat{N}(t,c)+{\hat{N}}_{0}}\cdot \hat{\Delta }(t,c) & \text{if}\hat{\Delta }(t,c)\geq 0\\ \hat{\Delta }(t,c) & \text{otherwise.} \end{array}\right) \end{array}$$

The dose-response model *R* is estimated pointwise by (7) with $\hat{N}(t,c)={\hat{\alpha }}_{\text{tc}}$. The dose-response curve for the *R*-model $\hat{R}(t,c)$ is obtained using isotonic regression and linear interpolation between the pointwise estimates.

#### ***Estimation of summary statistics***

The estimates for *GI*_50_, *TGI*, and *LC*_48_ are obtained by the concentration *c* where $\hat{G}(t,c)$, equals 0.5, 0, and -1/48, respectively. The summary statistic *AUC*_0_ is estimated by the area under the curve where $\hat{G}(t,c)\geq 0$. To compute confidence intervals for the summary statistics the following parametric bootstrap algorithm is applied with the number of iterations equal to *J*

1. For *j* in 1:*J*

1) Generate *K* plate sets on basis of the pre-processing model fitted to each cell line.

2) Fit the pre-processing model without outlier detection.

3) Fit the growth model to the pre-processed absorbance measurements.

4) Calculate the growth inhibition on basis of the *G*-model.

5) Estimate the summary statistics *GI*_50_, *TGI*, *LC*_48_, and *AUC*_0_.

2. Estimate a confidence interval for each summary statistic by use of the 2.5% and 97.5% percentiles of the *J* estimates obtained in step 5).

A similar approach can be used to estimate summary statistics for the dose-response models *D* and *R* with summary statistics obtained by the concentrations where $\hat{D}(t,c)$ and $\hat{R}(t,c)$ equal e.g. 0.5, 0, and -0.5 and 0.75, 0.5, and 0.25, respectively.

#### ***Correction of background absorbance***

When the drug under investigation is coloured like e.g. doxorubicin or interacts with the MTS assay, the background absorbance measures are elevated for increasing drug concentrations. This elevation necessitates correction when estimating the cell count [12]. One method is to include a background control for each concentration of the drug. Such an approach, however, requires a large number of wells. Alternative one may create a number of background plates with a setup similar to the one used for evaluating the cell count but without seeding cells into them. Next, these plates are pre-processed as described in *Model-based pre-processing*, which results in measurements of the absorbance caused by the drug. Finally, the excessive absorbance caused by each concentration of the drug is subtracted from the raw absorbance. This is done as an initial step before the Statistical analysis workflow.

### The simulation study

We compare the three dose-response models *G*, *D*, and *R* with a simulation study where the cell count is estimated through absorbance measurements based on nine simulated cell line models specified in Table 1. For each cell line this table includes the doubling time *T*_0_ and the summary statistics *GI*_50_, *TGI*, and *LC*_48_.

**Table 1:** Characteristics of the used cell line models

**Cell line**	***T***_**0**_	***GI***_**50**_	***TGI***	***LC***_**48**_
Cell 1	60	-8.40	-8.03	-7.57
Cell 2	30	-8.18	-7.78	-7.47
Cell 3	15	-7.95	-7.49	-7.27
Cell 4	15	-7.72	-7.27	-7.05
Cell 5	30	-7.50	-7.11	-6.80
Cell 6	60	-7.28	-6.91	-6.44
Cell 7	15	-7.05	-6.59	-6.37
Cell 8	60	-6.82	-6.46	-5.99
Cell 9	30	-6.60	-6.21	-5.90

Doubling time *T*_0_ and summary statistics *GI*_50_, *TGI*, and *LC*_48_ for the nine simulated cell line models.

The drug is added in triplicates for *C *= 18 decreasing concentrations in 96 well culture plates. Further, three wells are used as untreated controls and three wells are used as background controls. In order to illustrate the time dependence the cell count is estimated at four time points with 24-hour intervals. The net growth of the cell lines are modelled using the differential equation (1) with $1/T_{c}^{†}$ modelled by the following five parameter logistic function:

(17) $$1/T_{c}^{†}=b-\frac{d+b}{{\left(1+\text{exp}(a(c-e))\right)}^{f}}$$

where *a *= 2, *b *= 1/5, *d *= 0, *f *= 1. The parameter *e* is specified such that the summary statistics shown in Table 1 are obtained.

Each cell line model is simulated with *N*_0 _= 10,000 cells seeded into each well for all four plates at time *t *= 0 together with the drug. In order to imitate laboratory conditions the MTS assay is assumed added at time points $t_{1}^{\prime }=0,\;t_{2}^{\prime }=24,\;t_{3}^{\prime }=48,\text{and}\;t_{4}^{\prime }=72$ hours and the absorbance is measured after *t*_*inc *_= 2 hours. Consequently, the cell counts are generated at $t_{1}=t_{1}^{\prime }+t_{\text{inc}}/2=1,\;t_{2}=25,\;t_{3}=49,\text{and}\;t_{4}=73$. In order to attain absorbance measurements the proportionality factor *γ* in (10) is set equal to 0.4/10,000 for all nine cell line models.

In order to investigate the dose-response model *G*’s capability of estimating the summary statistics *GI*_50_, *TGI*, and *LC*_48_, noise is added to the system. To mimic real data this is done according to absorbance model (11) with parameters chosen in concordance with the estimates obtained for the B-cell cancer cell line panel introduced later.

For each cell line model the plate specific background absorbance *β*_*kt *_is drawn from a lognormal distribution with mean *μ*_*β *_= -0.8 and standard deviation *σ*_*β *_= 0.13; the plate specific multiplicative error *δ*_*kt*_is likewise drawn from a lognormal distribution with mean *μ*_*δ *_= 0 and standard deviation *σ*_*δ *_= 0.38. The parameters *μ*_*β*_, *μ*_*δ*_, *σ*_*β*_, and *σ*_*δ *_are respectively chosen as the mean and standard deviation of the log transformed estimates for *β* and *δ* obtained for the B-cell cancer cell line panel. Finally, the technical variation *ε*_*ktcl *_is drawn from a mean zero normal distribution with heteroscedastic variance $|\delta_{\text{kt}}\alpha_{\text{tc}}+\beta_{\text{kt}}{|}^{2\xi }\sigma_{\epsilon }^{2}$ where *ξ *= 1.42 and *σ*_*ε *_= 0.074 are chosen as the medians of the estimates for *ξ* and *σ*_*ε *_obtained for the B-cell cancer cell line panel.

The *Statistical analysis workflow* is used to obtain estimates of the summary statistics *GI*_50_, *TGI*, and *LC*_48_ associated with each cell line for 1,000 simulated datasets. Finally, the mean bias, standard deviation, and mean square error (MSE) are calculated for each cell line model and time point.

### The NCI60 cancer cell line panel

The cell line screen NCI60 is utilised to quantify the effect of a cell line’s doubling time on the *GI*_50_-value obtained by the dose-response model *D* in real data. Pharmacological data generated in the screen and modelled by the *D*-model is available online for 49,450 different compounds: http://dtp.nci.nih.gov. In this study we apply all compounds available in the September 2012 release that are tested at least three times on more than half the cell lines and for which half of the tested cell lines are affected by the drug. These criteria are satisfied for 1,699 different compounds.

The growth inhibition data is averaged for all experiments that are not already summarised by the mean. Next, the *G*-model is calculated by use of the transformation (8). For the dose-response models *G* and *D* the summary statistics *GI*_50_ and ${\text{GI}}_{50}^{D}$ are estimated using isotonic regression.

The association between the cell lines’ doubling time *T*_0_ and the summary statistics *GI*_50_ and ${\text{GI}}_{50}^{D}$ is determined using Pearson’s correlation coefficient for all 1,699 compounds. In order to determine whether or not the transformation causes a significant reduction in the correlation a paired t-test is used.

The reduction in the aforementioned correlation is further illustrated for doxorubicin and the drug with the greatest change. For these compounds the summary statistics *GI*_50_ and ${\text{GI}}_{50}^{D}$ are plotted against the doubling time *T*_0_, and the Pearson’s correlation coefficient is calculated.

### The B-cell cancer cell line panel

A doxorubicin dose-response screen of 14 Diffuse Large B-cell Lymphoma and 12 Multiple Myeloma cell lines is used to illustrate the proposed Statistical analysis workflow. The origin of the cell lines is as listed: KMM-1 and KMS-11 were obtained from JCRB (Japanese Collection of Research Bioresources). AMO-1, DB, KMS-12-PE, KMS-12-BM, LP-1, MC-116, MOLP-8, NCI-H929, NU-DHL-1, NU-DUL-1, OPM-2, RPMI-8226, SU-DHL-4, SU-DHL-5, and U-266 were purchased from DSMZ (German Collection of Microorganisms and Cell Cultures). FARAGE, HBL-1, OCI-Ly3, OCI-Ly7, OCI-Ly19, RIVA, SU-DHL-8, and U2932 were kindly provided by Dr. Jose A. Martinez-Climent (Molecular Oncology Laboratory University of Navarra, Pamplona, Spain). Finally, Dr. Steven T. Rosen generously provided MM1S.

The identity of the cell lines was verified by DNA barcoding performed every time a cell line was thawed and brought into culture. In brief, PCR analysis of left over traces of genomic DNA in 0.2 ng/*μ*l extracted RNA from cell lines was used as template in a multiplex PCR amplifying specific repeated DNA regions using the AmpFISTR Identifiler PCR amplification kit (Applied Biosystems, CA, USA). A fragment analysis of the amplified PCR products was performed by capillary electrophoresis (Eurofins Medigenomix GmbH, Applied Genetics, Germany). The resulting FSA file was analysed using the Osiris software (http://ncbi.nlm.nih.gov/projects/SNP/osiris) confirming the identity of the cell lines.

#### ***B-cell cancer cell lines and culture conditions***

The cell lines were cultured under standard conditions at 37° C; in a humidified atmosphere of 95*%* air and 5*%* CO _2_ with the appropriate medium (RPMI1640 or IMDM), fetal bovine serum (FBS), and 1*%* penicillin/streptomycin. The cell lines were maintained for a maximum of 20 passages to minimize any long-term culturing effects. Penicillin/streptomycin 1%, RPMI1640, IMDM and FBS were purchased from Invitrogen.

#### ***Dose-response experiments***

**Figure 4:**
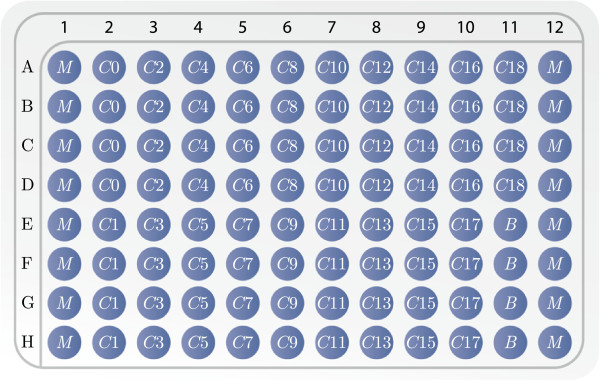
**Culture plate layout. **Wells labelled: *M* contain medium alone, *C*0 contain cell culture with salt water added at time $t_{1}^{\prime }$, *C*1,…,*C*18 contain cell culture with drug dilutions added at time $t_{1}^{\prime }$, and *B* contain only medium with salt water added at time $t_{1}^{\prime }$. The drug dilutions are given as 2-fold dilutions from *C *18 = 10 *μ*g/ml, *C *17 = 5 *μ*g/ml down to *C *1 = 763 · 10^-7^*μ*g/ml.

Doxorubicin was added in quadruplicates for *C *= 18 decreasing concentrations using the 96 well plate setup shown in Figure 4. All wells marked with a *C* in the table were seeded with cells and doxorubicin was added 24 hours later. Proliferation assays were performed using the CellTiter 96^®^; AQueous one Solution Reagent Cat no. G3580 (Promega, Madison WI, USA). The plates were incubated for *t*_*inc *_= 2 hours and absorbance was estimated at 492 nM using the Optima-Fluostar (BMG LABTECH). For plate 1, the reagent was added immediately after drug exposure and for plate 2, 48 hours later. With this approach the estimates of the cell count were obtained at approximately *t*_1 _= 1 hour, and *t*_2 _= 49 hours. To achieve high reproducibility, the entire experiment was repeated at least thrice. In order to avoid border effects only non-border wells were used for the subsequent analysis, whereby absorbance measurements are available in triplicates.

Doxorubicin is a coloured agent which was accounted for according to *Correction of background absorbance*. Using the corrected absorbance measurements, the dose-response model *G* and time independent summary statistics *GI*_50_, *TGI*, *LC*_48_, and *AUC*_0_ were estimated according to the established *Statistical analysis workflow*. The outlined bootstrap algorithm was applied to estimate 95% confidence intervals for the summary statistics with the number of iterations *J *= 200.

### Model check

Since different drugs have different action mechanisms one should investigate whether or not the proposed differential equation models data adequately well. This is, however, not possible if the dose response experiment has not been conducted for more than two time points. Here the experiment was expanded to include five time points $t_{1}^{\prime }=0$, $t_{2}^{\prime }=12$, $t_{3}^{\prime }=24$, $t_{4}^{\prime }=36$, $t_{5}^{\prime }=48$. Each of the five plates are configured with the same setup as that described in section *Dose-response experiments* with *t*_*inc *_= 2 hours. This approach gives estimates of the cell counts at approximately *t*_1 _= 1, *t*_2 _= 13, *t*_3 _= 25, *t*_4 _= 37, *t*_5 _= 49 hours. The experiment was repeated thrice. The differential equation model is fitted to the data using only *t*_1 _and *t*_5_. By plotting the data for all time points together with the fitted model it is possible to observe whether or not the model fits adequately well or whether a more advanced model is necessary.

## Results

### The simulation study

**Figure 5:**
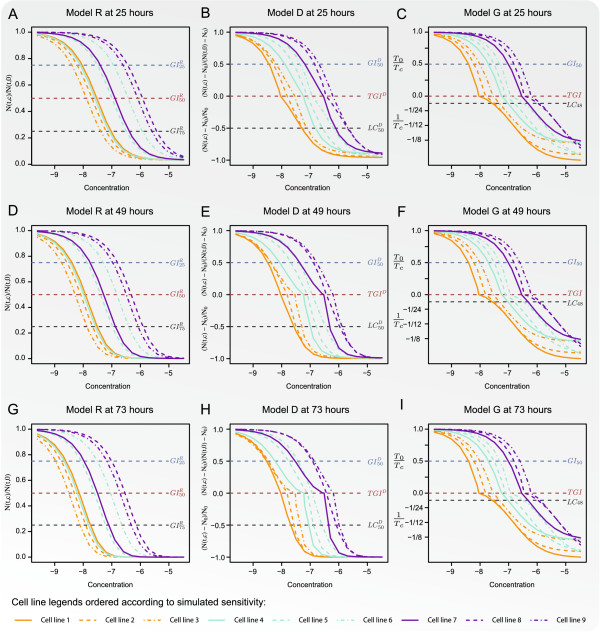
**The dose-response curves for the three dose-response models. **The dose-response curves for the *R*-model are shown in Panels **A**, **D**, and **G**, for the time points 25, 49, and 73 hours, respectively. Similarly for the *D*- and *G*-models Panels **B**, **E**, and **H** and **C**, **F**, and **I** show the dose-response curves obtained for time points 25, 49, and 73 hours, respectively.

Dose-response curves for the nine cell line models, described in *Methods*, are shown in Figure 5 for the dose-response models *G* (5), *D* (6), and *R* (7) and time points *t*_1 _= 25, *t*_2 _= 49, and, *t*_3 _= 73 hours.

The effect of the drug was modelled to be constant through time. However, according to both the *R*- and *D*-models the cell lines’ sensitivity toward the drug increased with time.

More specifically, for the *R*-model this is illustrated in Panels A, D, and G of Figure 5 which depict the obtained dose-response curves for the three time points. For each cell line model the dose-response curves had the same sigmoidal shape for all time points. As shown in (7) the *R*-model is indifferent towards the cell line doubling time which entailed that the drug sensitivity increased equivalently for each cell line model as the drug exposure time was prolonged. Furthermore, cell line model 1 was simulated as the most sensitive with a *GI*_50_-value of -8.4 log10(mmol/ml). However, for all time points, the *R*-model evaluated it as the fourth most sensitive, surpassed by cell line models 2, 3, and 4 which were simulated with *GI*_50_-values of -8.18, -7.95, and, -7.72 log10(mmol/ml), respectively.

For the *D*-model the increase in sensitivity was related to the growth rate of the cell line such that the order of the cell lines’ sensitivity interchanged through time. This is illustrated in Panels B, E, and H where the dose-response curves obtained by the *D*-model are shown for the three time points. The cell line models 3, 4, and 7 and 1, 6, and 8 were respectively fast and slowly growing. Accordingly, the increase in sensitivity through time was much more pronounced for cell line models 3, 4, and 7 than for 1, 6, and 8. In particular the fast growth rate of cell line model 7 and slow growth rate of cell line model 6 caused the obtained ${\text{GI}}_{50}^{D}$-values to interchange throughout the three time points.

The cell lines 1, 2, and 3 were simulated with *GI*_50_-values of -8.40, -8.18, and, -7.95 log10(mmol/ml), respectively; however, the ${\text{GI}}_{50}^{D}$-values obtained by the *D*-model were indistinguishable in Panel H. Additionally, the sensitivity level for cell line models 6 and 7 were reversed such that cell line 7 was evaluated to be more sensitive to the drug than cell line 6.

This implied that the summary statistics obtained by the *R*- and *D*-models were biased and relative to the cell lines’ sensitivity they were ordered incorrectly. Time independent summary statistics obtained by the *G*-model equalled those shown in Table 1.

The dose-response models *G* and *D* are continuous everywhere and differentiable everywhere except at the *TGI*-value. The latter results in the singularity occurring at that value for both functions. Since the *R*-model is continuous and differentiable everywhere such singularities do not occur for this model.

For each cell line model, 1,000 independent datasets were simulated with a culture plate and noise setup as outlined in *Methods*. The mean bias, standard deviation, and MSE for the summary statistics *GI*_50_, *TGI*, and *LC*_48_ obtained by the outlined statistical analysis workflow applied to the *G*-model are shown in Table 2 for the nine cell line models and three time points. The *Statistical analysis workflow* combined with the *G*-model was capable of producing unbiased estimates of the time independent summary statistics. The standard deviation and MSE were generally smallest for fast growing cell lines and decreased as the drug exposure time was prolonged.

**Table 2:** Summary of the simulation study

	**Mean bias**				**Standard deviation**				**MSE**		
	***GI***_**50**_	***TGI***	***LC***_**48**_		***GI***_**50**_	***TGI***	***LC***_**48**_		***GI***_**50**_	***TGI***	***LC***_**48**_
**Cell line 1, *T***_**0**_** = 60**											
Time 25	0.01	0.04	0.07		0.49	0.49	0.22		0.24	0.24	0.05
Time 49	-0.07	-0.06	0.00		0.36	0.36	0.14		0.13	0.14	0.02
Time 73	-0.05	-0.05	-0.01		0.25	0.25	0.11		0.06	0.07	0.01
**Cell line 2, *T***_**0**_** = 30**											
Time 25	-0.10	-0.08	-0.01		0.41	0.41	0.24		0.17	0.17	0.06
Time 49	-0.04	-0.04	-0.02		0.18	0.19	0.13		0.03	0.04	0.02
Time 73	-0.02	-0.02	-0.01		0.11	0.12	0.09		0.01	0.01	0.01
**Cell line 3, *T***_**0**_** = 15**											
Time 25	-0.05	-0.04	-0.02		0.21	0.25	0.21		0.05	0.06	0.05
Time 49	-0.01	-0.01	0.00		0.08	0.11	0.10		0.01	0.01	0.01
Time 73	-0.02	-0.01	0.00		0.07	0.09	0.08		0.01	0.01	0.01
**Cell line 4, *T***_**0**_** = 15**											
Time 25	-0.02	0.00	0.01		0.20	0.22	0.20		0.04	0.05	0.04
Time 49	-0.01	0.00	0.00		0.09	0.11	0.10		0.01	0.01	0.01
Time 73	-0.04	-0.04	-0.03		0.10	0.11	0.11		0.01	0.01	0.01
**Cell line 5, *T***_**0**_** = 30**											
Time 25	-0.08	-0.06	0.00		0.43	0.43	0.23		0.19	0.19	0.05
Time 49	-0.03	-0.03	-0.01		0.17	0.18	0.12		0.03	0.03	0.01
Time 73	-0.03	-0.03	-0.01		0.12	0.13	0.09		0.02	0.02	0.01
**Cell line 6, *T***_**0**_** = 60**											
Time 25	-0.07	0.00	0.07		0.69	0.63	0.23		0.47	0.40	0.06
Time 49	-0.08	-0.06	0.00		0.41	0.38	0.14		0.18	0.15	0.02
Time 73	-0.04	-0.03	0.00		0.25	0.23	0.10		0.06	0.06	0.01
**Cell line 7, *T***_**0**_** = 15**											
Time 25	-0.05	-0.03	-0.02		0.22	0.24	0.20		0.05	0.06	0.04
Time 49	-0.02	-0.01	0.00		0.09	0.11	0.10		0.01	0.01	0.01
Time 73	-0.10	-0.11	-0.04		0.22	0.23	0.10		0.06	0.06	0.01
**Cell line 8, *T***_**0**_** = 60**											
Time 25	-0.10	-0.03	0.06		0.73	0.65	0.22		0.54	0.42	0.05
Time 49	-0.14	-0.09	-0.02		0.49	0.41	0.15		0.26	0.18	0.02
Time 73	-0.06	-0.05	-0.01		0.29	0.27	0.11		0.09	0.08	0.01
**Cell line 9, *T***_**0**_** = 30**											
Time 25	-0.09	-0.06	0.00		0.46	0.44	0.23		0.22	0.20	0.05
Time 49	-0.04	-0.03	-0.01		0.17	0.19	0.12		0.03	0.04	0.02
Time 73	-0.02	-0.02	-0.01		0.11	0.12	0.08		0.01	0.01	0.01

The mean bias, standard deviation, and mean squared error of the summary statistics *GI*_50_, *TGI*, and *LC*_48_ obtained by the *G*-model for the 1,000 simulated datasets and the three time points.

### Model check

**Figure 6:**
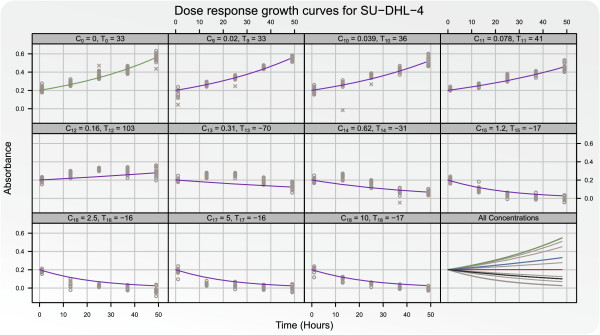
**The adequacy of the proposed differential equation model is checked.** Absorbance measurements Growth curves for the cell line SU-DHL-4 for five time points: *t*_1 _= 1, *t*_2 _= 13, *t*_3 _= 25, *t*_4 _= 37, and *t*_5 _= 49 hours are shown for the control *C*_0_ and under influence of the ten strongest concentrations of doxorubicin *C*_9_,…,*C*_18_. The growth curves are fitted using only the time points *t*_1_ and *t*_49_. The points correspond to the model-based pre-processed absorbance measurements. In the last panel the fitted growth curves for the cell line untreated (green) and for all ten concentrations (grey) are shown. In this panel the blue, red, and black curves correspond to the estimated growth curves at the summary statistics *GI*_50_, *TGI*, and *LC*_48_.

To check if the proposed differential equation models the dose response data adequately a time experiment was conducted. As an example of the model check the model-based pre-processed absorbance data for five different time points are shown in Figure 6 for the cell line SU-DHL-4. Model (12) was fitted using only the *t*_1 _= 1 and *t*_2 _= 49 hour time points. In this instance the model was found to fit the data adequately and that restricting the model fit to two time points yielded satisfactory results. However, it seems that the growth inhibition was underestimated for the large concentrations. This was the case for most cell lines and was a consequence of only using the 1 and 49 hour time points for estimating model (12).

### The NCI60 cancer cell line panel

**Figure 7:**
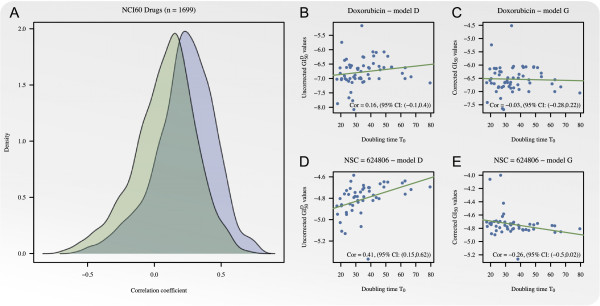
**Correction of the summary statistic *GI ***_**50**_**. **Panel **A**: Kernel estimated density functions of correlations between *T*_0_ and summary statistics ${\text{GI}}_{50}^{D}$ and *GI*_50_ for 1699 compounds obtained by the *D*-model (blue) and the *G*-model (green), respectively. Panel **B**: Correlation between *T*_0_ and the uncorrected summary statistic ${\text{GI}}_{50}^{D}$ obtained by the *D*-model for the single agent doxorubicin. Panel **C**: Correlation between *T*_0_ and the time independent summary statistic *GI*_50_ obtained by the *G*-model for doxorubicin. Panel **D**: Correlation between *T*_0_ and the uncorrected summary statistic ${\text{GI}}_{50}^{D}$ obtained by the *D*-model for NSC = 624806. Panel **E**: Correlation between *T*_0_ and the time independent summary statistic *GI*_50_ obtained by the *G*-model for NSC = 624806.

The NCI60 dose-response screen was used to illustrate how data obtained by the *D*-model can be transformed into the *G*-model by use of (8) and thereby be corrected for the cell line doubling time *T*_0_ and duration of the experiment. For the 1,699 compounds that satisfied the selection criteria the association between growth inhibition and growth rate was determined by Pearson’s correlation coefficient between *T*_0_ and the summary statistics *GI*_50_ and ${\text{GI}}_{50}^{D}$ estimated by the dose-response models *G* and *D*, respectively. Kernel estimated density functions of the correlations obtained using the two models are shown in Figure 7A. Because the bias associated with the *D*-model renders fast growing cell lines overly sensitive, the correlation between *T*_0_ and the summary statistic ${\text{GI}}_{50}^{D}$ was biased upwards. The average decrease of the aforementioned correlation, by adjusting for the cell line doubling time, was 0.145 (95 *%* CI: (0.14;0.15), p-value <0.001). More specifically, a significant negative correlation was found for 50 compounds using the *D*-model and 123 compounds using the *G*-model. In contrast, a significant positive correlation was found for 705 compounds using the *D*-model and only 278 using the *G*-model.

In Figure 7B to 7E the considered correlation is illustrated for the single agent doxorubicin and the drug giving rise to the greatest change in correlation by plotting the summary statistics ${\text{GI}}_{50}^{D}$ and *GI*_50_ against *T*_0_. For doxorubicin the correlation was 0.16, (95*%* CI: (-0.1,0.4)) for the uncorrected *D*-model and -0.03, (95*%* CI: (-0.28,0.22)) for the *G*-model. The drug with NSC number 624806 gave rise to the greatest change in correlation, specifically, from 0.41, (95*%* CI: (0.15,0.62)) for the uncorrected *D*-model to -0.26, (95*%* CI: (-0.5,0.02)) for the *G*-model.

The ten drugs with the greatest negative correlation between *T*_0_ and *GI*_50_ for model *G* have NSC numbers: 38721, 343513, 338720, 638850, 637404, 624807, 59729, 630684, 698673, and 353882. For model *D* the drugs were: 637404, 638850, 19994, 698673, 627666, 637603, 626734, 630684, 690134, and 37364. Out of these drugs four were found through both model *G* and *D*.

### The B-cell cancer cell line panel

**Figure 8:**
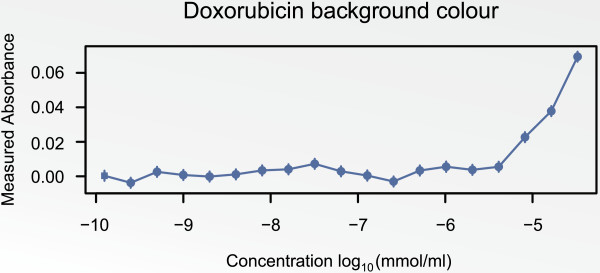
**Background absorbance measures as a function of the concentrations of doxorubicin.** The bars depict 95% CI’s associated with the absorbance.

A doxorubicin dose-response screen was used to illustrate the proposed *Statistical analysis workflow*. Doxorubicin is a coloured agent that elevates the absorbance measurements with increasing concentrations. In Figure 8 the background absorbance associated with each concentration is plotted. This was corrected for according to *Correction of background absorbance* prior to the application of the suggested pre-processing procedure.

**Figure 9:**
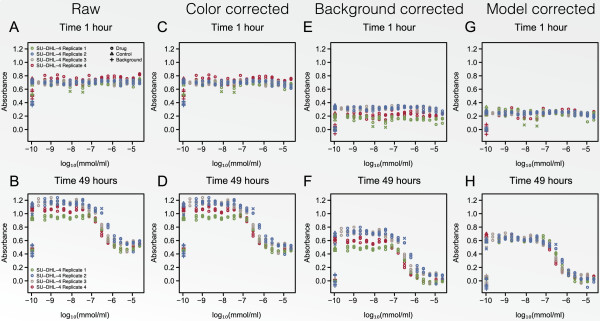
**The result of the pre-processing procedure is illustrated for the cell line SU-DHL-4.** The circles represent absorbance measures for the particular concentration at which it is plotted, the triangles represent the un-treated controls, the plusses represent background absorbance measurements, and, finally, the crosses illustrate outliers. The figure is divided into eight panels, where Panels **A**, **C**, **E**, and **G** show the results for time *t*_1 _= 1 hour and Panels **B**, **D**, **F**, and **H** for time *t*_2 _= 49 hours. Panels **A** and **B** show the raw absorbance measures for the four replicated experiments. The effect of the colour correction is shown in Panels **C** and **D**. Panels **E** and **F** illustrate the result of the conventionally applied background correction. Finally, the result of the model-based pre-processing procedure is illustrated in Panels **G** and **H**.

**Figure 10:**
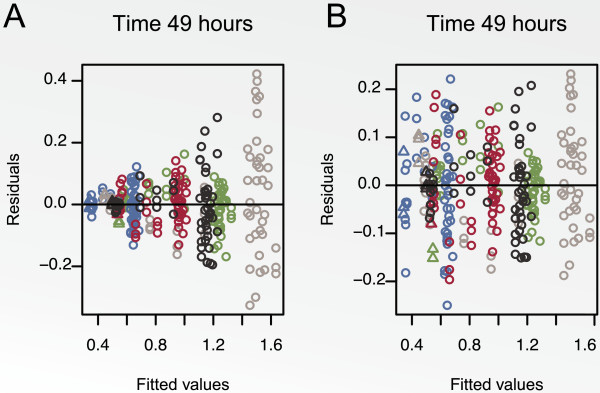
**Residual absorbance for cell line KMS-12-BM at 49 hours plotted against the pre-processed absorbance measure.** The three colours represent the triplicates. The triangles and circles represent background values and all other values, respectively. Panel **A** shows the result of ignoring the heteroscedasticity of the variance and Panel **B** shows the result of using the variance model.

In Figure 9 the result of the pre-processing procedure is illustrated for the cell line SU-DHL-4. Panels A and B show the raw absorbance measures for the four replicated experiments whereas the effect of the colour correction is shown in Panels C and D. In Panels E and F the results of the conventionally applied background correction are depicted. Finally, the result of the *Model-based pre-processing* is illustrated in Panels G and H. When comparing panels E and F to G and H we noticed that the mean absorbance was estimated with a considerable lower variance when the model-based pre-processing was used. A cross marks the measurements that are found to be outliers and for example two of the un-treated control measurements for plate 2 were deemed to be outliers as illustrated in Panel H. In panel H these measurements were clearly extreme values, however, prior to the model-based pre-processing this was not the case.For each cell line residual plots of the final pre-processing models were investigated to ensure that the absorbance model fitted the data reasonably well. For cell line KMS-12-BM the residual plot is illustrated in Figure 10. Panel A shows the residual plot obtained when the heteroscedasticity of the variance was ignored, whilst Panel B shows the residual plot when the variance model was fitted. These plots confirm that the variance model was capable of fitting the heteroscedastic variance observed in dose-response experiments.

**Figure 11:**
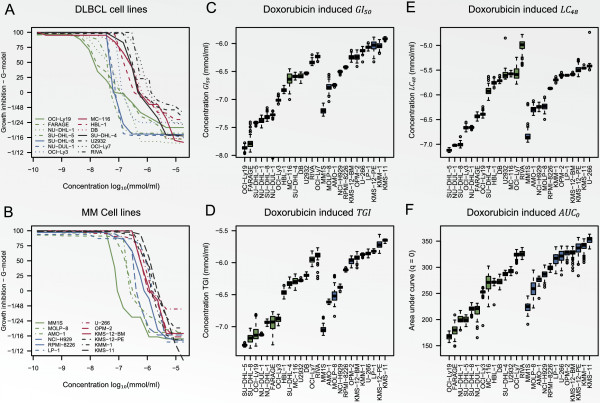
**Illustration of dose-response curves for the B-cell cancer cell line panel.** Panels **A** and **B** illustrate dose-response curves obtained by the *G*-model for the 14 DLBCL and 12 MM cancer cell lines. Panels **C**, **D**, **E**, and **F** depict boxplots of the bootstrapped summary statistics *GI*_50_, *TGI*, *LC *48, and *AUC*_0_, respectively. The green and blue colours are used for DLBCL and MM cell lines, respectively.

The dose-response curves obtained for the 14 DLBCL and 12 MM cancer cell lines are illustrated in Panels A and B of Figure 11. The first quadrant of the plots depicts the percentage growth for the treated cell line compared to the same cell line un-treated, e.g. the values 75, 50, and 25 were attained at the concentrations where the doubling time for the control was 75, 50, and 25% of that for the treated cell line, or equivalently, the growth rate of the treated cell line was 75, 50, and 25% of that for the un-treated cell line. The fourth quadrant depicts cell decay, e.g. the values -1/48, -1/24, and -1/16 were attained at the concentrations where the cell line population was halved in 48, 24, and 16 hours, respectively. None of the curves contained points where the treated cell line outgrew the controls, i.e. values greater than 100. This was an effect of forcing $1/T_{c}^{†}$ to be positive which was of great importance for the summary statistic *AUC*_0_. The estimated cell line doubling time and summary statistics *GI*_50_, *TGI*, *LC *48, and *AUC*_0_ are shown in Table 3 with associated 95% confidence intervals. The bootstrapped summary statistics *GI*_50_, *TGI*, *LC *48, and *AUC*_0_ are also illustrated by box plots in Panels C, D, E, and F of Figure 11.

**Table 3:** Summary statistics for the B-cell cancer cell line panel

**Cell Line**	***T***_**0**_		***G*-Model**			
	**Hours**		***GI***_**50**_	***TGI***	***LC***_**48**_	***AUC***_**0**_
**DLBCL**						
OCI-Ly7	24 (21;26)		-6.23 (-6.31;-6.18)	-5.96 (-6.05;-5.84)	-5.57 (-5.74;-5.31)	325 (310;332)
RIVA	68 (55;91)		-6.35 (-6.39;-6.27)	-5.87 (-6.18;-5.79)	-4.97 (-5.36;-4.82)	327 (316;337)
U2932	34 (33;35)		-6.53 (-6.56;-6.51)	-6.28 (-6.29;-6.25)	-5.60 (-5.68;-5.48)	295 (285;299)
DB	37 (35;39)		-6.58 (-6.65;-6.52)	-6.19 (-6.25;-6.15)	-5.71 (-5.76;-5.65)	276 (260;285)
SU-DHL-4	36 (34;39)		-6.59 (-6.64;-6.52)	-6.33 (-6.39;-6.24)	-5.92 (-6.05;-5.73)	289 (277;294)
MC-116	50 (41;70)		-6.64 (-6.87;-6.48)	-6.29 (-6.38;-6.20)	-5.57 (-5.62;-5.53)	272 (233;300)
HBL-1	46 (42;51)		-6.83 (-6.89;-6.78)	-6.46 (-6.55;-6.37)	-5.76 (-5.83;-5.68)	272 (266;277)
OCI-Ly3	91 (69;119)		-7.02 (-7.06;-6.94)	-6.89 (-6.96;-6.81)	-6.67 (-6.73;-6.59)	253 (244;259)
NU-DUL-1	48 (40;63)		-7.27 (-7.33;-7.21)	-7.13 (-7.14;-7.11)	-7.02 (-7.04;-7.00)	224 (191;231)
SU-DHL-8	85 (53;175)		-7.32 (-7.39;-7.21)	-7.19 (-7.32;-7.10)	-7.01 (-7.07;-6.93)	222 (207;231)
NU-DHL-1	29 (27;32)		-7.39 (-7.49;-7.29)	-6.94 (-6.98;-6.89)	-6.66 (-6.70;-6.63)	201 (184;213)
SU-DHL-5	32 (30;35)		-7.42 (-7.44;-7.40)	-7.29 (-7.32;-7.26)	-7.12 (-7.16;-7.08)	202 (189;209)
FARAGE	58 (49;72)		-7.79 (-7.93;-7.49)	-6.93 (-7.23;-6.80)	-6.44 (-6.52;-6.37)	180 (158;198)
OCI-Ly19	39 (36;45)		-7.87 (-7.95;-7.79)	-7.13 (-7.25;-6.83)	-6.39 (-6.46;-6.31)	167 (157;178)
**MM**						
KMS-11	48 (45;51)		-5.91 (-5.94;-5.88)	-5.65 (-5.68;-5.63)	-5.46 (-5.48;-5.44)	356 (340;361)
KMM-1	44 (40;52)		-6.03 (-6.13;-5.95)	-5.87 (-5.91;-5.85)	-5.70 (-5.74;-5.66)	346 (316;350)
KMS-12-PE	24 (20;29)		-6.04 (-6.24;-5.91)	-5.73 (-5.82;-5.61)	-5.50 (-5.61;-5.32)	340 (310;353)
LP-1	33 (30;36)		-6.07 (-6.12;-6.02)	-5.82 (-5.85;-5.80)	-5.60 (-5.62;-5.59)	316 (294;336)
U-266	48 (43;54)		-6.13 (-6.20;-6.06)	-5.86 (-5.91;-5.80)	-5.42 (-5.47;-5.37)	325 (299;339)
OPM-2	57 (47;71)		-6.24 (-6.33;-6.13)	-5.98 (-6.07;-5.91)	-5.61 (-5.64;-5.57)	329 (313;339)
KMS-12-BM	47 (42;57)		-6.25 (-6.33;-6.16)	-5.92 (-6.02;-5.85)	-5.59 (-5.61;-5.56)	331 (307;337)
RPMI-8226	30 (29;32)		-6.42 (-6.45;-6.40)	-6.11 (-6.14;-6.09)	-5.87 (-5.89;-5.85)	297 (292;306)
NCI-H929	28 (25;31)		-6.50 (-6.54;-6.48)	-6.39 (-6.42;-6.34)	-6.25 (-6.31;-6.17)	291 (270;300)
AMO-1	32 (30;34)		-6.74 (-6.76;-6.72)	-6.61 (-6.64;-6.58)	-6.30 (-6.40;-6.17)	269 (265;283)
MOLP-8	34 (21;48)		-6.78 (-6.93;-6.62)	-6.52 (-6.67;-6.27)	-6.23 (-6.37;-6.10)	253 (229;290)
MM1S	37 (26;51)		-7.20 (-7.29;-7.10)	-7.05 (-7.12;-6.95)	-6.87 (-6.94;-6.60)	227 (208;239)

The doubling time T_0_ and summary statistics *GI*_50_, *TGI*, *LC*_48_, and *AUC*_0_ for the dose-response model *G*.

## Discussion

In the present study a differential equation that models drug induced growth inhibition of human tumour cell lines was established. Based on this equation a novel model for summarising dose-response experiments was produced, that in combination with a statistical workflow, is capable of generating unbiased time independent summary statistics.

To determine if the differential equation is adequate for modelling real data a time experiment based on doxorubicin was conducted. The experiment included five time points of which only the first and last were used to fit the differential equation. The differential equation was found to model the data adequately, albeit the use of only two time points may lead to an underestimated drug efficiency for large doses. Since the differential equation was found adequate, a simulation study was performed to document the potential bias when using existing methods, and the robustness of the workflow. We deduced that under the proposed differential equation these summary statistics are biased estimators of growth inhibition so that the drug effect is amplified concurrently with increasing growth rate of the cell lines.

In Kondoh et al. [18] 40 representative anticancer drugs from the NCI60 screen were used to illustrate the association between cell line growth rate and drug sensitivity assessed by the *D*-model. They found the growth rate was positively correlated with drug sensitivity. We propose that this finding is partly caused by systematic bias induced by the experimental setup of the cell line screens. Since the difference between the treated and un-treated cell line will increase with time, the effect of the drug will seem greater for fast growing cell lines. We showed that by transforming data obtained by the *D*-model into the *G*-model the correlation between doubling time and drug resistance decreased significantly. We do not argue against drug resistance being associated with growth rate as the authors successfully discover and validate a potential new anticancer drug, we merely suggest that removing the design-based bias may lead to a range of new potential drugs to be investigated.

In order to illustrate the suggested workflow for dose-response experiments, a study of 26 cell lines tested for drug resistance at 18 different concentrations of doxorubicin was presented. The results illustrate that it is possible to gain realistic estimates of the variance of the growth inhibition characteristics, which is of great value in the application of dose-response studies.

### Practical considerations

Since the establishment of NCI60, dose-response screens of human tumour cell lines have been one of the most commonly used methods for discovering new anticancer drugs [2]. The approach has mostly been used to discover drugs that are potent in a considerable part of the tested cell lines originating from various tumour types. With this purpose in mind, the bias introduced by analysing cell lines with different doubling times has little or no influence on the conclusions. More recently, the cell line screens have been used to discover treatments that are only potent in a small proportion of the tested cell lines and hence in a small proportion of the cancer patients. Ignoring the doubling time of the cell lines may reduce the capability of discovering such drugs since slowly growing cell lines may appear resistant to the drug.

The issue of growth rate bias may be remedied by using cell lines with approximately the same doubling time or alternatively by conducting the experiments using individual time spans corresponding to a given number of doubling times for each cell line. Based on the latter approach Bracht et al. [19] took the doubling times into account by conducting the dose-response experiments such that each of the 77 cell lines was exposed to the drug for three cell line specific doubling times. For large cell line screens it is neither feasible to generate diverse panels consisting of cancer cell lines with similar doubling times nor is it practical to conduct each experiment for different time spans. The latter option is further complicated since it may not be possible to keep slowly growing cell lines in the exponential growth phase for several doubling times throughout the experiment. The models used in cell line screens NCI60 [3], JFCR39 [5], and CMT100 [2] are based on fixed drug exposure times. We established transformations of these models so that each cell line’s doubling time can be accounted for.

### Methodological considerations

Modelling the growth of a cell line exposed to an anticancer drug by the simple differential equation (1) facilitated a meticulous analysis of existing summary statistics for cell line based dose-response studies of growth inhibition. It may be possible to establish a differential equation that leads to either the *D* or *R* model. However, the authors have not been able to do so in an unblemished fashion. It is thus difficult to determine which assumptions must be met for the results of these models to be unbiased.

The differential equation was based on exponential cell growth which seems a reasonably assumption since all drug response assays strive toward using the exponential growth phase of the cell lines for the out-read window. Similarly, the rate for cells going into cell cycle arrest or death is assumed exponential and concentration dependent, partly due to computational convenience and partly because no obvious alternative is present. It should be emphasized the assumption of an exponential rate for cells going into cell cycle arrest or death induce a constant drug efficiency throughout the experiment. However, since different drugs induce growth inhibition by different mechanisms, the established differential equation (1) is oversimplified and may therefore model the growth of a cell line exposed to a drug inadequately. It would be interesting to establish more complex systems of differential equations of cell culture growth in combination with more measurements during drug exposure time [20-25]. This would allow estimation of drug induced growth inhibition with improved precision and hence increased biological understanding.

A model-based approach to pre-processing based on a nonlinear regression model was introduced. This model efficiently and simultaneously addresses a number of issues such as background absorbance correction, multiplicative seeding effects and heteroscedastic variance of absorbance measures. All are well-known nuisance effects in cellular/bacterial growth studies [11,26]. The modelling approach also facilitated outlier detection by residual analysis and standard model checks from regression theory [17]. The dose-response relationship was modelled by the growth curves arising from the solution to the posed differential equation. This lead to pointwise estimates of the dose-response curve of the *G*-model and interpolation of the curve was done by isotonic regression which is robust against outliers and model misspecifications [14,27].

Providing precision estimates of the growth inhibition characteristics in this complex setting is not straightforward, so parametric bootstrap of the nonlinear model of the absorbance measurements was used [28]. Alternatively the statistical delta method could have been applied [11,29]. Although feasible, this would have required complicated approximations by Taylor series expansions, and bootstrapping is generally considered to have superior small sample properties [30].

The dose-response model *R* (7) based on relative cell counts is very appealing due to its simplicity. Moreover, it is a smooth function so it is possible to fit parametric models to the dose-response curve of *R* which facilitates extrapolation. When extrapolation is necessary it is possible to fit a parametric model to the dose-response curve of *R*[31,32] and subsequently transform the result into the dose-response curve of *G* using (9). This approach facilitates estimation of time independent summary statistics by extrapolation.

## Conclusions

In this study we have shown that conventionally used dose-response models can give rise to biased summary statistics erroneously correlated to the growth rate of the cell lines. We have developed novel summary statistics of dose-response experiments that are applicable on existing data and independent of time under the proposed differential equation. Consequently, we expect that the present approach will be able to improve future drug evaluation studies.

## Competing interests

The authors declare that they have no competing interests.

## Authors’ contributions

All authors conceived the project. SF and MB designed the differential equation model and algorithms for estimating the associated coefficients with confidence intervals. The laboratory studies were designed by SF, MBL, JSB, MKK, KD, and MB and performed by MBL, JSB, and MKK. AS, MN, HEJ, and KD contributed reagents and materials used in the study. SF implemented algorithms and performed the computational experiments. All authors analyzed the results, were involved in manuscript preparation, and read and approved the final manuscript.
